# The Accuracy of Clinical Thermometers

**Published:** 1881-06

**Authors:** 


### EXCERPTA.

THE ACCURACY OF CLINICAL THERMOMETERS.—A "Card to the Medical Profession," from the *Winchester Observatory*, of Yale College, dated February 1, 1881, and signed by Mr. Leonard Waldo, the astronomer in charge, reads as follows: "The competition of business, coupled with the entire absence, up to this time, of any large observatory in this country paying special attention to thermometry, to which authoritative appeal could be made, has so affected the manufacture of thermometers for medical purposes, that it seems necessary to issue a card briefly indicating the errors commonly found to exist, and to explain why, in this case, the representations of the dealers may be at fault through the want of a proper understanding of the subtle errors to which medical thermometers are liable. Too great a desire to economise time, good material and skilled labor has led, in the making of thermometers, to the following faults: 1. The graduation is sometimes started from one point of the scale, near the normal, and the size of the capillary tube is guessed at. No upper point being fixed by the maker, the higher graduations may be erroneous to the extent of several degrees. 2. Too much air separating the index from the column of mercury causes the index to rise with a jerky motion; air above the index forces the index down when the thermometer is taken away from the body. In some thermometers errors from this cause amount to two degrees at high temperatures. 3. New thermometers increase their readings rapidly during the first months after manufacture, so that instruments which were right when made may change their indications as much as two degrees within a year. It will be seen that these errors are not such as the dealer can readily detect. Even in those cases where a dealer is provided with a standard thermometer with which comparisons could be made, it is a difficult matter to determine the errors of the standard itself, and the unsupported representations of dealers and druggists, therefore, though made in perfect good faith, cannot, from the nature of the case, afford the physician satisfactory evidence that any thermometer he may buy is not affected with errors, which in many instances under our observation have amounted to several degrees. Following the example of the Royal Society's observatory at Kew, at which during the past year upward of five thousand thermometers were examined, this observatory has established a department to which any physician or other person may send thermometers by mail or express, and upon the payment of a small fee receive certificates of their exact errors. The facilities are such that there is no good reason why physicians should not buy their thermometers furnished with the Yale certificates by the dealer; in those cases where no certificate is furnished the uncertainty may amount to two degrees. It should be remembered that thermometers which the physician has had in his possession for many months are certain to have had the requisite seasoning, and therefore an old thermometer with a recent certificate is more valuable than a new one, or one about whose age there is doubt. The observatory has been called upon within three months to certify about seven hundred thermometers from various parts of our country; the results of this work have demonstrated the gross inaccuracy of the cheaper clinical thermometers as commonly sold, and seem to render expedient the publication of this card, calling the attention of physicians to these errors and the great difficulty of detecting them except with the appliances of an observatory devoted to this work."—*New York Medical Journal.*

